# THE IMPORTANCE OF EMOTIONAL CAPITAL ACROSS THE LIFESPAN: WHY THE LITTLE MOMENTS IN MARRIAGE MATTER

**DOI:** 10.1093/geroni/igz038.1048

**Published:** 2019-11-08

**Authors:** Lisa Neff, Courtney Walsh, Jennifer Beer

**Affiliations:** University of Texas at Austin, Austin, Texas, United States

## Abstract

Throughout a marriage, couples will share countless ordinary moments together, such as laughing together or engaging in leisure activities. Although these moments may seem trivial in isolation, research suggests that accumulating small positive moments together helps couples build emotional capital, which serves as an essential resource for protecting marriages from the harmful consequences of relationship challenges. This study explored whether emotional capital may buffer couples not only from the negative effects of relational stressors, but also from the negative effects of life stressors encountered outside the relationship in a sample of younger (age 30-45) and older (age 60+) married couples. Drawing from theories of socioemotional expertise, we also examined whether the buffering effects of emotional capital may be stronger for older adults. One hundred forty-five couples completed a 21-day daily diary task assessing shared positive experiences with the partner, negative partner behaviors, marital satisfaction, life stress, and mood. Spouses who generally accrued more shared positive moments with their partner across the diary days maintained greater marital satisfaction on days of greater partner negativity compared to spouses who accrued fewer positive moments. Moreover, spouses who generally accrued more shared positive moments with their partner across the diary days also reported lower levels of negative mood on days in which they experienced more life stress compared to spouses who accrued fewer shared positive moments; in both cases, the buffering role of emotional capital was significantly stronger for older adults. All results held when adjusting for relationship length and general marital happiness.

